# Outcomes and experiences of families with children with type 1 diabetes on insulin pumps through subsidised pump access programs in Western Australia

**DOI:** 10.3389/fendo.2023.1173559

**Published:** 2023-06-08

**Authors:** Vivian R. Fu, Kathleen Irwine, Kirsty Browne-Cooper, Craig E. Taplin, Timothy W. Jones, Elizabeth A. Davis, Mary B. Abraham

**Affiliations:** ^1^ Medical School, University of Western Australia, Perth, WA, Australia; ^2^ Department of Endocrinology and Diabetes, Perth Children’s Hospital, Perth, WA, Australia; ^3^ Children’s Diabetes Centre, Telethon Kids Institute, University of Western Australia, Perth, WA, Australia; ^4^ Division of Paediatrics, Medical School, University of Western Australia, Perth, WA, Australia

**Keywords:** subsidised insulin pump, experiences, financial cost, type 1 diabetes, glycaemic outcome

## Abstract

**Introduction:**

In Australia, access to insulin pump therapy for children with type 1 diabetes (T1D) is predominantly restricted to families with private health insurance. In an attempt to improve equity, additional subsidised pathways exist which provide pumps to families with reduced financial resources. We aimed to describe the outcomes and experiences of families with children commenced on pumps through these subsidised pathways in Western Australia (WA).

**Methods:**

Children with T1D in WA who did not have private health insurance and received pumps from the subsidised pump programs between January 2016 and December 2020 were included. Study 1 was designed to review glycaemic outcome. A retrospective analysis of HbA1c was conducted in the whole cohort and in children who commenced pump after the first year of diagnosis to exclude the impact of the partial clinical remission phase following diagnosis. HbA1c at baseline, and six, 12, 18 and 24 months after pump initiation were collected. Study 2 was designed to review experiences of families commenced on pumps through subsidised pathway. A questionnaire designed by the clinical team was distributed to parents *via* an online secure platform to capture their experiences.

**Results:**

Of the 61 children with mean (SD) age 9.0 (4.9) years who commenced pump therapy through subsidised pump programs, 34 children commenced pump therapy after one year of diagnosis of T1D. The median (IQR) HbA1c (%) in 34 children at baseline was 8.3 (1.3), with no statistically significant change from baseline at six months [7.9 (1.4)], 12 months [8.0 (1.5)], 18 months [8.0 (1.3)] or 24 months [8.0 (1.3)]. The questionnaire response rate was 56%. 83% reported intention to continue pump therapy, however 58% of these families did not have avenue to acquire private health insurance. Families expressed inability to procure private health insurance due to low income and unreliable employment and remained largely unsure about the pathway to obtain the next pump.

**Discussion:**

Children with T1D who commenced insulin pump therapy on subsidised pathways maintained glycaemic control for two years, and families favored pumps as a management option. However, financial limitations persist as a significant barrier to procure and continue pump therapy. Pathways for access need to be assessed and advocated.

## 1 Introduction

Advancements in diabetes technology with insulin pump therapy, continuous glucose monitoring (CGM) systems and automated insulin delivery systems have revolutionised diabetes care. These systems are increasingly adopted as standard care in clinical practice (1) with international pediatric guidelines advocating for equal access to technology for all people with diabetes (2). However, the uptake is influenced by multiple factors. In general, although it is largely believed that health care professionals are more likely to support the use of these systems, discrepancies exist with health care professional (HCP) bias in offering management choices to families (3) compounded by issues of staff resources (4) and skill in adopting technology. Although it is known that insulin pump therapy is an established management option in children diagnosed with type 1 diabetes (T1D) and is associated with overall better glycaemic outcomes (5, 6), access to these devices is limited by high cost and HCP recommendation to commence pump therapy is largely influenced by health care coverage or insurance (7) with heterogeneity in access to diabetes technology globally (8) While a few countries have government-funded programs (3, 8–10) which provide access to pumps and/or CGM, the availability of these devices in many parts of the world is restricted to families self-funding these technological devices. There remains a large disparity in the use of diabetes technologies with multiple barriers in procuring access and maintaining the use of the device in the socioeconomically disadvantaged communities (11).

The Australian health care system supports management of T1D through provision of free needles and syringes with a subsidy to insulin and consumables (eg glucose monitoring strips and pump supplies) with a copayment through the Pharmaceutical Benefits Scheme and National Diabetes Subsidy Scheme respectively. Furthermore, since 2017, children and young adults <21 years receive full subsidy for CGM devices. However, there are no supports for accessing insulin pumps.

In Australia, full payment for an insulin pump with four-year warranty costs $7,000 to 10,000 (12). Thus, most families are reliant on private health insurance with high premium policies to accommodate the cost of the device (13–17). Families without private health insurance have limited avenues to fund a pump, especially in those with reduced financial capacity. Some families interested in pump therapy access pumps through Government means-tested subsidised Insulin Pump Program (IPP), administered by Juvenile Diabetes Research Foundation (JDRF) (18); however, availability is limited in terms of both total number of pumps available and device type. This pathway, by itself, is thus not sufficient to support all families with reduced financial resources. Hence, in an attempt to improve equity of technology access to children with T1D in Western Australia (WA), the Perth Children’s Hospital (PCH) Pump Program funded by a PCH Foundation Grant was established in 2010. Access to a fully subsidised insulin pump was made available for families without private health insurance and dependent on income support from the Government. Prior to commencement, interested families received education on the advantages and disadvantages of pump therapy by their usual clinical team, were informed of ongoing out-of-pocket monthly costs of $30-40 for consumables (pump reservoirs, cartridges, and infusion sets) and the requirement for internet and computer access. Families were supported by the diabetes team through structured pump education workshops and regular follow-up to facilitate transition to pump use together with the same educational modules and appointments provided to children commencing pumps funded through insurance. They were also encouraged to plan private health insurance to finance a new pump following the four-year warranty period if they wished to continue pump therapy, as the PCH Pump Program was designed and funded to cover the cost of one pump device.

This study was designed to explore the glycaemic outcomes and experiences of families with a child with T1D who received an insulin pump through these subsidised pathways.

## 2 Materials and methods

### 2.1 Study design

To address the aims of the study, we had two study designs. Study 1 was a retrospective, longitudinal, observational analysis of glycaemic outcomes and Study 2 was a cross-sectional survey evaluating experiences of the family.

The study was conducted at Perth Children’s Hospital, a tertiary paediatric statewide diabetes centre that provides local and outreach care to all children with T1D in WA under 18 years of age.

Children with T1D (age < 18 years) commenced on subsidised insulin pumps through the JDRF and PCH Pump Programs between January 2016 and December 2020 were included. Children who commenced insulin pumps through private health insurance were excluded. Demographic and clinical data were collected from the WA Children’s Diabetes Database, a prospective database with clinical, anthropometric and glycaemic data collected during clinic visits. The socioeconomic background was based on lack of private health insurance and assessed using the Socio-Economic Indexes for Areas (SEIFA) decile (19) according to family’s postcode of residence. Formal approval was obtained from the project governance body for quality improvement activities (quality improvement activity number 42141).

### 2.2 Study 1: Glycaemic outcome

A retrospective analysis of HbA1c was conducted in children who commenced pumps after the first year of diagnosis to exclude impact of the partial clinical remission phase following diagnosis. Data of children commenced on insulin pumps between January 2016 and December 2020 were included to permit up to 24-month analysis of HbA1c of the cohort. This timeframe was chosen to better reflect families on established pump therapy. For children who had discontinued pump therapy, the last available HbA1c while on pump therapy was included. HbA1c was done as part of usual quarterly clinical care by point of care testing. HbA1c at baseline, six, 12, 18 and 24 months after pump initiation were collected, ± one month from the exact time point of interest. Children who commenced pumps during the first year of diagnosis were analysed separately.

### 2.3 Study 2: Participant experiences

An anonymous questionnaire designed by the clinical research team was administered *via* an online secure platform to parents of children who commenced pump therapy *via* the PCH Pump Program as a Quality Improvement Project. The questionnaire gathered chronological experiences of families before, during and after starting pumps. Specifically, it explored the ease of T1D management with insulin pump (20), financial impact associated with pump use, satisfaction with the PCH Pump Program, and family’s future management intentions (questionnaire attached as supplementary material). Those who had ceased pump therapy had additional questions about reasons for discontinuation. Responses were encouraged by email and phone call reminders sent to parents over a six-month period from October 2021 to March 2022.

### 2.4 Data analysis

Demographic and clinical data are reported as descriptive statistics in the form of median (IQR), mean (SD) and proportions (%). Change in HbA1c from baseline at each time point (six, 12, 18 and 24 months) was analysed using paired t-tests. The response rate to the questionnaire was reported as a percentage of total number of families invited to participate in the study.

## 3 Results

### 3.1 Demographics

61 children from families without private health insurance and dependent on income support from the Government received insulin pumps through subsidised insulin pump program. This pathway contributed to 12.5% of the clinic cohort on pump therapy. This cohort had a mean (SD) age of 9.0 (4.9) years, diabetes duration of 2.4 (3.1) years and baseline HbA1c 65 (13) mmol/mol or 8.1 (1.2) % with 38% living in areas of low socioeconomic status. 54 children received pumps through the PCH Pump Program and seven through IPP. 55 (90%) children were also using a CGM device. No children were on closed loop therapy. 10 participants ceased pumps and returned to insulin injections; the mean (SD) duration of insulin pump use in this group was 19.2 (12.3) months. The demographic data of the clinic cohort characterised by the treatment modality are summarised in **Table 1**.

**Table 1:** Characteristics of children on insulin injections and insulin pumps through private health insurance and subsidised pumps.

	Multiple Daily Insulin Injections	Insulin pumps *Via* Private Health Insurance	Insulin pumps Via Subsidised Pump Program
n	434	424	61
Age* (years)	12.2 (4.0)	12.5 (4.0)	9.0 (4.9)
Sex, n (%)			
Males	234 (54)	210 (49.5)	36 (59)
Females	200 (46)	214 (50.5)	25 (41)
Diabetes Duration* (years)	3.2 (3.3)	5.9 (3.8)	2.4 (3.1)
HbA1c* %	8.4 (1.9)	8.2 (1.5)	8.1 (1.2)
mmol/mol	68 (20)	66 (16)	65 (13)
SEIFA decile^#^ n (%)			
1-3	103 (24)	83 (20)	23 (38)
4-7	155 (35)	158 (37)	22 (36)
8-10	159 (37)	162 (38)	16 (26)
N/A	17 (4)	21 (5)	0
Location n (%)			
City and major regional areas	339 (78)	301 (71)	42 (69)
Regional areas	78 (18)	102 (24)	19 (31)
N/A	17 (4)	21 (5)	0 (0)
Ethnicity** n (%)			
Oceanian (Australia, NZ)	289 (66.6)	231 (55)	41 (67)
Northwest European	21 (4.8)	33 (7.8)	5 (8)
Southern and Eastern European	7 (1.6)	10 (2.4)	0 (0)
North African and Middle Eastern	6 (1.4)	4 (0.9)	4 (7)
Sub Saharan African	16 (3.7)	13 (3.1)	2 (3)
South-East Asian	8 (1.8)	2 (0.5)	0 (0)
North-East Asian	4 (0.9)	3 (0.7)	0 (0)
Southern and Central Asian	13 (3)	11 (2.6)	1 (2)
People of Americas	2 (0.5)	2 (0.5)	0 (0)
Not available	68 (15.7)	115 (27.1)	8 (13)
**CGM use** n (%)	386 (89)	400 (94)	55 (90)

*Values in Mean (SD),^#^SEIFA: Socio-Economic Indexes for Areas (SEIFA) decile. A lower decile represents relatively greater disadvantage, while a higher decile indicates relative lack of disadvantage.

**Self-reported ethnicity is categorised according to the Australian Bureau of Statistics’ (ABS) Australian Standard Classification of Cultural and Ethnic Groups (ASCCEG), Second Edition, 2011.

### 3.2 Glycaemic outcome

**Figure 1:**
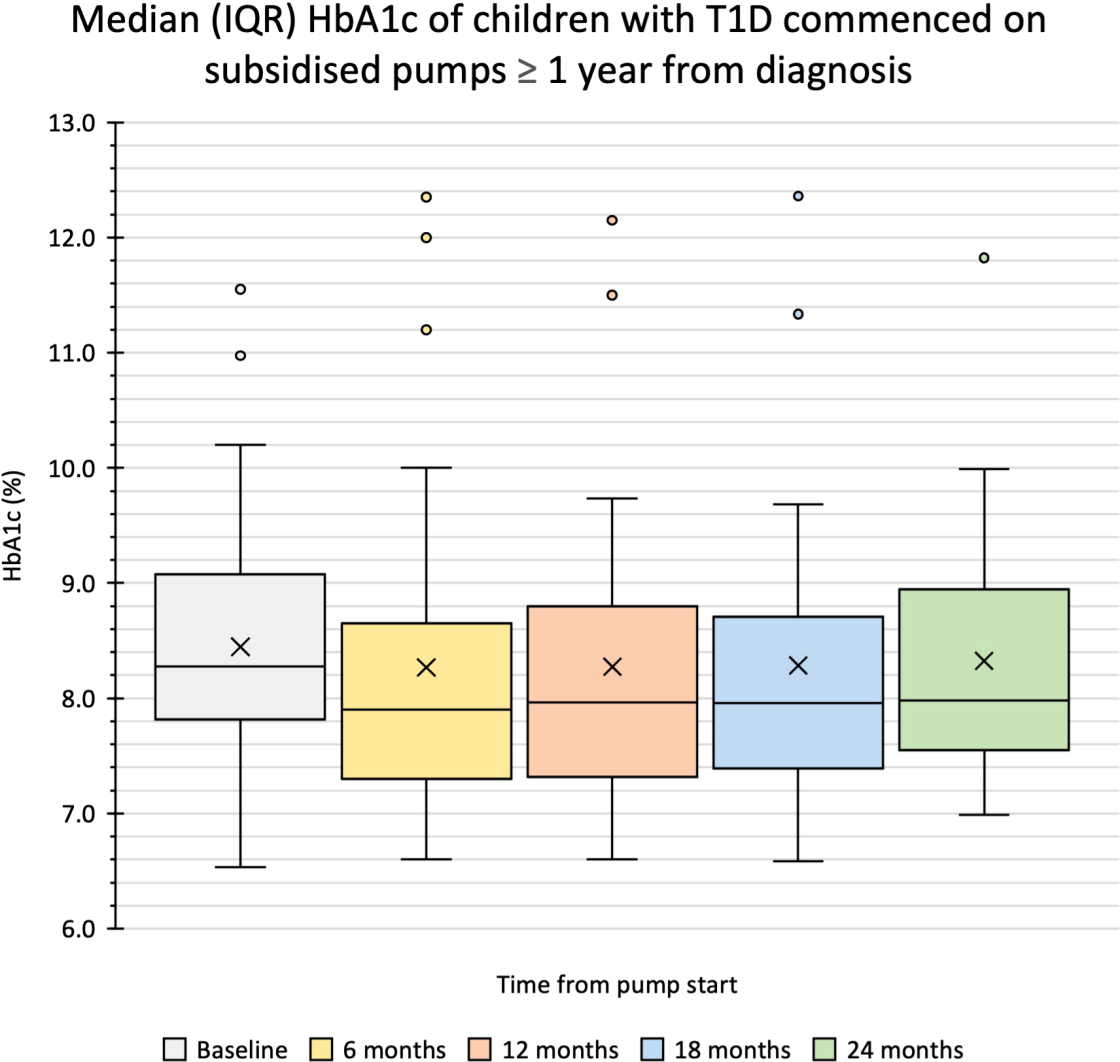
Median (IQR) HbA1c of children with T1D commenced on subsidised pumps ≥ 1 year after diagnosis. Box and whisker plot shows the median, the first and third quartile, the minimum and maximum numbers and the outliers.

Glycaemic outcomes were evaluated in 59 children; two were excluded as they were using insulin pumps through the subsidised pump programs prior to the study period. In this cohort, 34 children, with a mean (SD) age of 11.2 (3.1) years and diabetes duration 4.0 (3.3) years commenced insulin pump therapy more than one year after T1D diagnosis with a median (IQR) HbA1c of 67 (14) mmol/mol or 8.3 (1.3) % at baseline. The HbA1c was 63 (15) mmol/mol or 7.9 (1.4) % at six months, 64 (16) mmol/mol or 8.0 (1.5) % at 12 months, 64 (14) mmol/mol or 8.0 (1.3) % at 18 months, and 64 (14) mmol/mol or 8.0 (1.3) % at 24 months with no significant difference in change in HbA1c at any of these timepoints from baseline (**Figure 1**, **Table 2**). In children who commenced insulin pump therapy within the first year of diagnosis [n = 25, aged 5.6 (4.9) years, diabetes duration 3.9 (3.6 months)], a similar pattern was noted as shown in **Table 2**.

**Table 2:** Glycaemic outcomes of children with T1D commenced on subsidised pumps.

	Started pump ≥ 1 year diagnosis				
	n	HbA1c Median (IQR)		Mean change from baseline, % (95% CI)	p
		Mmol/mol	%		
Baseline	34	67 (14)	8.3 (1.3)		
6 months	33	63 (15)	7.9 (1.4)	-0.1 (-1.5, 1.3)	0.57
12 months	32	64 (16)	8.0 (1.5)	-0.1 (-1.4, 1.2)	0.68
18 months	31	64 (14)	8.0 (1.3)	-0.1 (-1.4, 1.2)	0.15
24 months	23	64 (14)	8.0 (1.3)	-0.1 (-1.1, 1.0)	0.22
	Started pump <1 year diagnosis				
	n	HbA1c Median (IQR)		Mean change from baseline, % (95% CI)	p
		Mmol/mol	%		
Baseline	23	62 (9)	7.8 (0.8)		
6 months	25	58 (13)	7.5 (1.2)	-0.1 (-1.2, 1.0)	0.33
12 months	24	63 (15)	7.9 (1.4)	0.1 (-1.1, 1.3)	0.57
18 months	23	65 (22)	8.1 (2.0)	0.4 (-1.0, 1.8)	0.18
24 months	21	67 (16)	8.3 (1.5)	0.7 (-0.7, 2.1)	0.18
	Total				
	n	HbA1c Median (IQR)		Mean change from baseline, % (95% CI)	p
		Mmol/mol	%		
Baseline	57	64 (15)	8.0 (1.4)		
6 months	58	63 (14)	7.9 (1.3)	-0.1 (-1.4, 1.2)	0.35
12 months	56	64 (16)	8.0 (1.5)	0 (-1.2, 1.2)	0.95
18 months	54	65 (17)	8.1 (1.6)	0.1 (-1.2, 1.4)	0.95
24 months	44	66 (16)	8.2 (1.5)	0.2 (-1.0, 1.4)	0.75

### 3.3 Participant experiences

All 54 families with children who commenced on insulin pump therapy through the PCH Pump Program were invited to complete a questionnaire. The demographic data are summarized in **Table 3**. The response rate was 56% (n = 30), one response was partially completed while the remaining 29 responses were fully completed. 87% (n = 26/30) of parents reported the discussion to start a pump was initiated by healthcare staff while three families actively sought pumps.

**Table 3:** Characteristics of children commencing pumps through the PCH Pump Program grouped by participation in the questionnaire.

		Parent completed questionnaire		Did not complete questionnaire	
Total, n (%)	54	30 (56%)		24 (44%)	
Sex, n (%)	Female	18	(60%)	17	(71%)
	Male	12	(40%)	7	(29%)
Age (years)	Mean (SD)	9.5	(4.5)	8.9	(5.4)
Diabetes duration (years)	Mean (SD)	2.4	(3.1)	2.4	(2.7)
Baseline HbA1c, Mean (SD)	mmol/mol	64	(11)	68	(16)
	%	8.0	(1.0)	8.4	(1.5)
SEIFA decile, n (%)	1-3	11	(37%)	11	(46%)
	4-7	6	(20%)	11	(46%)
	8-10	13	(43%)	2	(8%)
Location, n (%)	City and major regional areas	21	(70%)	16	(67%)
	Regional areas	9	(30%)	8	(33%)

83% (n = 24/29) of parents reported that their child was still using the insulin pump at the time of the survey; two pumps were out of warranty and two families did not know the warranty status of their pump. Of those using the pump, 63% (n = 15/24) rated T1D management ‘easy/relatively easy’, 29% (n = 7/24) rated it ‘neutral’ and 8% (n = 2/24) rated it ‘difficult/relatively difficult’. 29% (n = 7/24) reported additional financial stress associated with starting pump therapy. Notably, all of these families reported the $30-40 monthly cost of pump consumables remained an ongoing burden, other reported costs impacting families included the cost of taking out health insurance to cover future pump access, internet access and equipment (alcohol wipes, hypoallergenic tapes, batteries).

Five families reported that their child had ceased insulin pump therapy. The most common reason was the child’s dislike of *wearing* the device (n = 3), other reasons included dislike *using* the device (n = 1), incompatibility with hobbies (n = 1) and warranty expiry of subsidised pump with lack of private health insurance to cover for next pump (n = 1).

Overall, 79% (n = 23/29) were either satisfied or very satisfied with the PCH Pump Program. 83% (n = 24/29) of parents expressed their child intends to continue to use pump therapy for future diabetes management. Ten families accessed/intended to access private health insurance to acquire the next pump, however 58% (n = 14/24) of this group did not have avenues to afford private health insurance. Open-ended question exploring how they intend to proceed with pump therapy in the future revealed that most families were unsure (n=11), the reported social difficulties included low income, unreliable employment, being a single parent, or being a special needs family. These responses are demonstrated in **Table 4**.

**Table 4:** Financial limitations voiced by carers of children using subsidised Pump Programs.

Carer responses to open question “How will you go about obtaining a second pump?”		
“I am hoping my medical issues will improve allowing me to gain reliable employment soon though. For now, we will just try to care for this pump so we can use this pump for as long as possible.”	“Not sure as not financially secure.”	“I have been working and saving to buy one outright, I cannot afford health insurance being a single parent.”
“Hopefully she will be able to afford her own private health insurance, when she gets a job.”	“We are a family with many special needs and that impacts us financially … private health insurance would be very unrealistic on our current budget. We are so fortunate our child received a pump because we couldn’t have afforded it.”	“Not sure as situation hasn’t changed. May have to see if I can afford health insurance in the future.”

## 4 Discussion

This report highlights a center-based approach to improve access to pump therapy and thus reduce inequities in diabetes technology in a statewide pediatric diabetes service in Australia. Although overall, the U.S-based Type 1 diabetes Exchange (T1DX), the German/Austrian Diabetes Prospective Follow-up (DPV),the international SWEET and the Australasian Diabetes Data Network (ADDN) registries have reported an increased adoption of technological devices over the last decade (10, 21–23), inequities in device use by race and socioeconomic status have also surfaced (24–26) in conjunction with differences in medical reimbursement patterns (8, 27). The lower rates of diabetes technology and higher HbA1c reported in youth from lower socioeconomic status at higher risk of poor metabolic outcomes (28–30) raise concerns that this group remains vulnerable in an era of evolving diabetes technologies (31). Furthermore, uptake differed by degree of socioeconomic deprivation and ethnicity even with access to government-funded insulin pump program (3), highlighting the need to review social determinants of health (32) to better understand the recommendations to inform practice. These differences in the access and uptake of technology potentially risk increasing disparities in glycaemic outcomes and quality of life, highlighting the need to address pathways to mitigate this risk.

More than 50% of children in the clinic were on insulin pump therapy at the time of the study. The clinic practice is to offer pump therapy to interested families with 61 of the 485 families on pumps having received devices through the subsidised insulin pump programs. These children and their families from a socioeconomically disadvantaged background, although interested in pump therapy, were unable to access it due to the financial burden associated with procuring the device. Majority were Caucasian families and the location data provides additional insight into this cohort’s characteristics. More families lived in relatively low (38%) and middle decile (36%) areas as compared to high decile areas (26%) within WA, reflecting greater disadvantage (19). The subsidised pump program made insulin pumps accessible to these families, of which 31% were from regional areas. It is also worthwhile to note that 90% of the children were on CGM, adopting this technology after the Australian Federal Government scheme provided subsidy to CGM devices to children and young adults <21 years in 2017. This highlights that families embraced technology when access was made available to them in conjunction with support from the diabetes team ensuring continued maintenance of therapy.

The study explored glycaemic outcomes for 24-months of transitioning from insulin injections to pump therapy. After excluding youth with diabetes duration less than one year and thus any impact of the honeymoon period, children commenced on insulin pump therapy through this program showed stable glycaemic control and no evidence of change in HbA1c from baseline to 24 months. The impact of psychosocial stressors impacting diabetes management remains high in this vulnerable cohort, who require more targeted approach as indicated on initial social work assessment. We have previously shown in our population-matched cohort that children with T1D using pumps had better long-term glycaemic control than those using injections (33). It showed a mean HbA1c difference of 0.4% (p = 0.04) over a ten-year follow-up period and demonstrated improved HbA1c in the initial period after pump start. This current study was underpowered to detect a difference of this magnitude, however the size and direction of the point estimates from 8.3% to 8.0% after 24 months of pump therapy are consistent with the previous study. It is noteworthy that children from this cohort potentially have outcomes comparable to the general population.

The study also explored the parent-reported experiences from families who had the opportunity to commence their child on insulin pump through the PCH pump program. The questionnaire responses showed most parents were satisfied and felt well supported. The easier and more challenging aspects of diabetes management reported by parents of children who received subsidised pumps were similar to a qualitative study in families who accessed pumps *via* private health insurance (20). Importantly, our cohort voiced financial challenges which may impede their ability to continue insulin pump use. The cost of private health insurance for device access and the ongoing costs of pump consumables, which are only partially subsidised by the Australian Government National Diabetes Service Scheme, highlights the financial needs for a family interested in pump therapy. Notably, a majority of families remained unable to afford pump therapy years after commencing a pump through the subsidised pathway. Families expressed inability to procure private health insurance with low income and unreliable employment and remained largely unsure about the pathway to obtain the next pump. Prolonged use of current pump, working and saving to buy a pump and hoping that the child will be able to afford the pump when older were some of the strategies highlighted by the respondents. Thus, many families remain financially vulnerable and one-time funding for diabetes technology is neither a long-term nor a stable solution to address systemic inequities in access to pump technology in Australia.

The strength of this study is that it reviews the glycaemic outcomes and highlights the parent-reported experiences from pathways designed to improve technology access to families with limited financial abilities. The main limitation of this study is the retrospective study design in a single centre with a small sample size with missing data due to nature of data collection. Hence, this study was restricted to the time frame between 2016 and 2022 although the subsidised pathways were in place prior to the chosen study duration. Data on parental education, employment and income were not available. Ideally, participants using insulin pumps are matched to those on injections for glycaemic analysis, however, this was difficult to achieve given the demographics of the cohort accessing subsidised pumps. Furthermore, it would be useful to review a larger dataset including families who accessed JDRF insulin pump program across other paediatric centres in Australia. The questionnaire used for qualitative assessment was not externally validated and response rates may be impacted by the lack of access to technologies in this cohort and may not adequately reflect the characteristics of the cohort with the possibility that the non-responders were likely to be less satisfied.

In conclusion, the financial limitations contribute greatly to the inequity of pump access within Australia. Subsidised pump programs provide pump access to those without private health cover; however, their availability is restricted. It is also important to note that in countries with government funding like Canada and New Zealand, although there is an increased pump uptake, the support and maintenance of therapies may be further influenced by complex socioeconomic characteristics highlighting the need for a continued role in ongoing efforts to ensure equitable access (9, 10). The universal access to insulin pumps, especially in the context of closed loop therapy adopted as standard care, still remains pivotal and is a critical step for ensuring equity of advanced therapies to individuals with T1D.

## Data availability statement

The raw data supporting the conclusions of this article will be made available by the authors, without undue reservation.

## Ethics statement

The study received approval from GEKO (Governance Evidence Knowledge Outcomes) Committee, Department of Health, Western Australia (Quality Improvement Activity Number 42141). Informed consent was obtained from all patients' legal guardian/next of kin prior to their data being stored on clinical and research database (Western Australian Children’s Diabetes Database, WACDD) (HREC 2012051EP).

## Author contributions

MA contributed to the conception and supervision of all aspects of the study. VF helped with data collection, analysis and wrote the manuscript. MA, KB-C and VF contributed to design of the questionnaire. MA edited the subsequent drafts of the manuscript. All authors contributed to the article and approved the submitted version.
