# Supplementary material for: Outcomes and experiences of families with children with type 1 diabetes on insulin pumps through subsidised pump access programs in Western Australia
Source: Front Endocrinol (Lausanne). 2023 Jun 8;14:1173559. doi: 10.3389/fendo.2023.1173559 (PMC10286577; doi:10.3389/fendo.2023.1173559)
Supplement: Supplementary file 1 [file DataSheet_1.docx]

Supplementary Material

Outcomes and experiences of families with children with type 1 diabetes on insulin pumps through a pump access program in Western Australia

Vivian R Fu, Kathleen Irwine, Kirsty Browne-Cooper, Craig E Taplin, Timothy W Jones, Elizabeth A Davis, Mary B Abraham^*^

*** Correspondence:** Dr Mary B Abraham: [mary.abraham@health.wa.gov.au](mailto:mary.abraham@health.wa.gov.au)

**Questionnaire provided to parents of children who commenced subsidised insulin pump therapy.**

**1 Before starting pumps**

How did you first hear about the insulin pump?

Please select all applicable:

- Clinic
- Internet/media
- Family member
- Friends
- Others using insulin pumps
- Unsure
- Other, please specify: *open text*

How did the discussion of subsidised pump program begin?

Please select:

- Clinician brought up the topic in clinic
- You/family brought up the topic in clinic
- Unsure
- Other, please specify: *open text*

**2 Pump experiences**

Is your child still using a subsidised insulin pump?

Please select:

- - Yes (see below)
  - No

Why did your child stop using the insulin pump?

Please select all those applicable:

- - Pump warranty ended and unable to access private health insurance for ongoing use
  - Don’t like *using* the insulin pump
  - Don’t like *wearing* the insulin pump
  - Other, please specify: *open text*

If Yes:

1. Is your child’s insulin pump still under warranty?

Please select:

- - Yes
  - No

1. How do you find managing type 1 diabetes using the insulin pump?

Please rate:

- 1. Easy
  2. Relatively easy
  3. Neutral
  4. Relatively difficult
  5. Difficult

Please specify reason for your answer: *optional open text*

1. How do you find having to upload pump information before attending diabetes clinics?

Please rate:

- 1. Easy
  2. Relatively easy
  3. Neutral
  4. Relatively difficult
  5. Difficult

Please specify reason for your answer: *optional open text*

1. Has starting on the insulin pump added extra financial strains on the family?

Please select:

- - Yes

Through which areas has starting insulin pump affected you financially?

Please select all that apply:

- - - $30-40 monthly consumable costs
    - Cost of internet access
    - Cost of computer access
    - Other, please specify: *open text*
  - No

**3 Future plans**

Does your child intend to continue using insulin pump in the future for diabetes management?

Please select:

1. Yes

Has your financial situation changed to allow your child to get private health insurance for a pump? (Please select)

- - Yes
  - No
  - Prefer not to answer

If no private health insurance: How will you go about obtaining a second pump?

- Please specify: *open text*

1. No

Please select all that apply:

- - Pump warranty ended and unable to access private health insurance for ongoing use
  - Don’t like using the insulin pump
  - Don’t like wearing the insulin pump
  - Other, please specify: *optional open text*

1. Unsure

**4 Overall satisfaction**

Overall, how satisfied are you with the experience of starting an insulin pump through the PCH Pump Program?

Please rate:

1. Very unsatisfied
2. Unsatisfied
3. Indifferent
4. Satisfied
5. Very satisfied
